# Improving Sensitivity of a Micro Inductive Sensor for Wear Debris Detection with Magnetic Powder Surrounded

**DOI:** 10.3390/mi10070440

**Published:** 2019-07-01

**Authors:** Liankun Liu, Liang Chen, Saijie Wang, Yi Yin, Dazhuang Liu, Sen Wu, Zhijian Liu, Xinxiang Pan

**Affiliations:** 1 College of Marine Engineering, Dalian Maritime University, Dalian 116026, China; 2 School of Science, Dalian Maritime University, Dalian 116026, China; 3 College of Electronic and Information Engineering, Guangdong Ocean University, Zhanjiang 524088, China

**Keywords:** micro inductive sensor, sensitivity, magnetic powder, wear debris, improvement

## Abstract

The inductive detection of wear debris in lubrication oil is an effective method to monitor the machine status. As the wear debris is usually micro scale, a micro inductive sensor is always used to detect them in research papers or high-tech products. However, the improvement of detection sensitivity for micro inductive sensors is still a great challenge, especially for early wear debris of 20 μm or smaller diameter. This paper proposes a novel method to improve the detection sensitivity of a micro inductive sensor. Regarding the magnetic powder surrounding the sensor, the magnetic field in the core of the sensor where the wear debris pass through would be enhanced due to the increased relative permeability. Thus, the inductive signal would be improved and the detection sensitivity would be increased. It is found that the inductive signal would linearly increase with increasing the concentration of the magnetic powder and this enhancement would also be effective for wear debris of different sizes. In addition, the detection limit of the micro inductive sensor used in our experiment could be extended to 11 μm wear debris by the proposed method.

## 1. Introduction

The failure of a running machine may result in high safety risk or economic loss. Researchers have been working to develop measures to prevent such failures. An effective way is on-line monitoring of the machine’s status, followed by timely maintenance which has attracted a lot of attention [1]. Over the last decades, several on-line monitoring methods, such as vibration patterns [2,3], thermal analysis [4] and oil particulate analysis [5], have been proposed and developed. While the vibration patterns and thermal analysis can provide vital information concerning the current status of the machine, the oil particulate analysis can forecast early abnormal wear and provide a prognosis of pending machinery failure that is very important for marine engines and aircraft engines. Wear debris detection is key to the oil particulate analysis. A variety of methods for on-line wear debris detection, such as capacitive detection [6,7], acoustic detection [8,9], color extraction method [10,11] and inductive sensor, have been developed and they were reviewed in a recent article [12]. Among these methods, the inductive sensor shows great advantages such as simple structure, low cost, ability to differentiate ferrous and nonferrous debris and so on [13,14,15,16,17,18].

Based on the principle of electromagnetic induction, the inductive sensor induces a magnetic field when a current flows through the coil. The wear debris will interact with the magnetic field when it passes through the coil and the current will change. Consequently, the wear debris can be detected by sensing the changes. One challenge for inductive sensor to forecast abnormal wear is improving the detection sensitivity as the size of early wear debris is very small (always below 20 μm). It is fact that an inductive sensor with a very small inner diameter, which is comparable to the early wear debris, could detect them. However, this method has two disadvantages for the inductive sensor, low throughput and easy to block. Regardless, many researchers work on improving the inductive sensor detection sensitivity without reducing the inner diameter, mainly following two directions. The first is reducing the level of the noise and second is improving magnitude of the signal.

Bozchalooi et al. [19] and Luo et al. [20] proposed their work on reducing the level of the noise. Usually, the wear debris signal is similar to a sine wave while the environment interference is composed by random noises and some periodical waveforms caused by mechanical vibration or AC power. As a result, the output of the sensor combined with these waveforms is non-stationary. They proposed a two-stage de-noising scheme. In the first stage, a wavelet-based adaptive subband filtering technique is applied to remove the vibration-related interferences. The outputs of the adaptive filters are then thresholded in the second stage to remove the background noise mainly caused by the wiring and measurement system flaws.

Another way to improve the sensitivity is to increase the magnitude of the signal. Du et al. [21,22] analyzed the magnetic field generated by a coil with different ratios of length to diameter and they proposed that a low length-to-diameter ratio could benefit sensitivity. Hong et al. [23] designed a sensor structure based on a radial magnetic field which could detect 200 μm debris within a pipe with a diameter of 20 mm. After that, they designed a symmetrical structure with permanent magnets to further optimize strength and uniformity. Through this improvement, 83 μm debris could be detected within a pipe with a diameter of 12 mm under a flow rate of about 20 L/min [24], which is valuable for practical applications. Du et al. also developed another method to improve the sensitivity using the inductance–capacitance (LC) resonance. With the method, 20 μm ferromagnetic debris and 55 μm diamagnetic debris can be detected in the glass tube with inner diameter of 1 mm [25]. Zhu et al. [26] presented a wear debris sensor with ferrite cores for online monitoring which is capable of detecting 50 μm ferrous debris in 7 mm diameter fluidic pipes. 

In this paper, we proposed a novel method to improve the sensitivity of the sensor where the sensor is surrounded by magnetic powder. We experimentally studied the effect of concentration of magnetic powder on the detection signal and the effect of the wear debris size was also examined. Finally, the detection limit of the proposed method was explored. This method is effective for detection of the early wear debris.

## 2. Sensor Design and Detection Principle 

**Figure 1:**
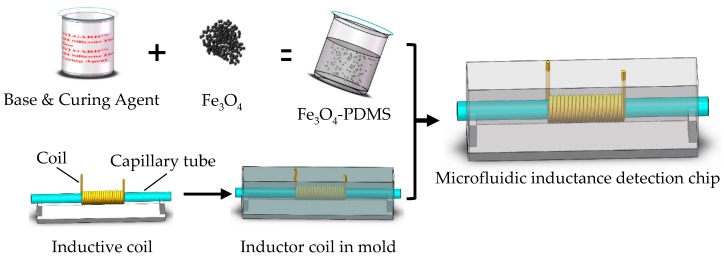
The fabrication of the 3-D solenoid coil with magnetic powder surrounded.

A three-dimension (3-D) solenoid coil, shown in Figure 1, was used as the micro inductive sensor. To build the 3-D solenoid coil, initially a small steel wire (600 µm in diameter) with smooth surface polish was prepared. Then, a 50 turns 3-D solenoid coil was built by carefully winding the fine copper wire (65 µm in diameter, with a thin insulation) around the small steel bar using automatic hot air winder (YZE-1200, Dongguan YinZhuoEn Precision Automation Co., Ltd., Dongguan, China). A capillary tube (with inner diameter 300 μm and outer diameter 500 μm), inserted into the coil carefully, was used as the flow channel of wear debris or oil sample.

To increase the magnetic conductivity of the environment outside the sensor, some magnetic powder was placed around the coil by the following method. A small amount of magnetic powder (800 mesh, Shenzhen Youci Technology Co. LTD, Shenzhen, China) was mixed with polydimethylsiloxane (PDMS) (Sylgard 184, Dow Corning, Midland, MI, USA) with different weight ratios, from 0 to 70%. After degassing the mixture in a vacuum oven (Model 280A, Fisher Scientific, Hampton, NH, USA) at −26 inHg, the mixture was poured into a mold in which the micro inductive sensor with a capillary tube was fixed. The mold was kept in a vacuum oven (Model 280A, Fisher Scientific, Hampton, NH, USA) at 60 °C for 2 h. The mold was taken out after the mixture solidification. Finally, the finished chip has a thickness of 6 mm.

The basic inductance of the chip used in our experiment was measured and the results are shown in Table 1. It should be noted that the basic inductance of the chip would be increased as the concentration of the magnetic powder increased. This is mainly caused by the enhanced relative magnetic permeability of the environment.

An alternating current was applied across the 3-D solenoid coil, which induced an alternating magnetic field in the sensor. The impedance of the sensor can be calculated by

(1) Z=R+jωL

where *j*^2^ = −1, *Z* is the impedance of the coil, *R* and *L* are the resistance and inductance of the coil, respectively, and ω is the angular frequency of the alternating current. The impedance *Z* is determined by the alternating magnetic field. When the oil containing metallic wear debris passes through the sensor, the magnetic field is changed due to the influence of the wear debris. As a result, the impedance *Z*, as well as the resistance *R* and the inductance *L*, will also change.

(2) $$L\approx N^{2}\mu_{0}\mu_{r}\left(\frac{D}{2}\right)\left(\ln\frac{8D}{d}-2\right)$$

where *N* is the number of turns, $\mu_{0}$, $\mu_{r}$ is permeability of free space and relative permeability. *D* and *d* is loop diameter and wire diameter, respectively. So, the detection signal of the wear debris would be affected by the permeability of the space where the solenoid coil is located.

## 3. Experiments and Discussions

### 3.1. Sample Preparations and Experimental Procedure

Wear debris was attached on a fiber using micro motion platform. The sphere-shaped wear particles were purchased from Qinghe Chuangying Metal Material Co., Ltd. (Xingtai, China). The diameter of the wear debris was measured by optical microscope. The wear debris attached on the fiber can be used repeatedly. Thus, we can use the same wear debris in comparative experiments so that the error caused by wear debris feature can be neglected.

**Figure 2:**
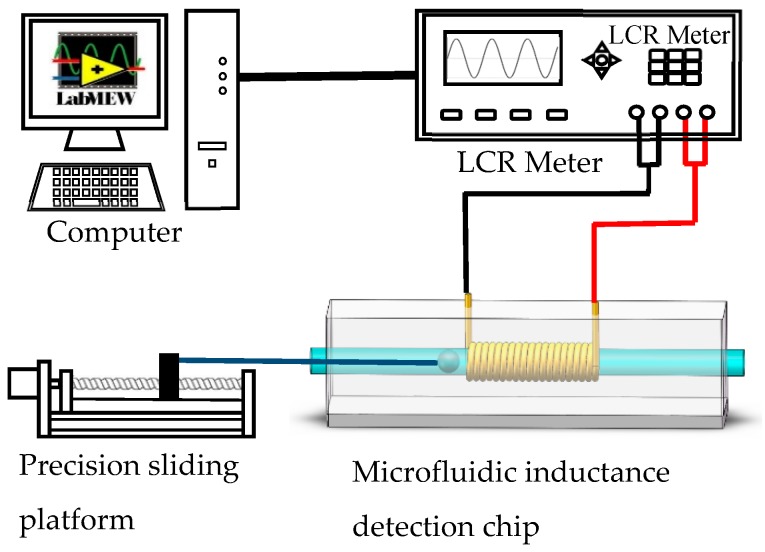
The detection system for single wear debris.

The experimental system is illustrated in Figure 2. It is composed of a micro inductive sensor, an inductance (L), capacitance (C), and resistance (R) meter (Agilent E4980A Precision LCR Meter, Agilent Technologies Inc., Bayan Lepas, Malaysia), a precision sliding platform and a computer. Single wear debris and oil sample were used to demonstrate our method. When the wear debris passed through the coil, an inductance pulse signal appeared. The signals measured by the LCR meter were transmitted to the computer through the LabVIEW^®^ (LabVIEW 2010, National Instruments, Austin, TX, USA). The difference between the peak value of each signal and the average value of the noise band was used as the magnitude of the pulse signal.

For single wear debris experiment, the spherical metal particles were fixed at one end of the nylon fiber while the other end of which was fixed on the precision sliding platform as shown in Figure 2. A stepping motor derived the platform to move linearly at a speed of 25 mm/s. The inductive detection of each wear debris was repeated 10 times and the average value of the pulse magnitude was calculated as the final value. The experimental data was processed in Microsoft Excel. For the oil sample experiment, a small amount of wear debris, size ranging from 74 to 88 μm, was mixed with oil (Alexia S6, Shell Ltd., Hague, Dutch). This sample was pumped into the capillary tube by micro pump at a flow rate of 0.12 mL/min.

### 3.2. Results and Discussions

#### 3.2.1. The Influence of Magnetic Powder Concentration on the Detection Signal

**Figure 3:**
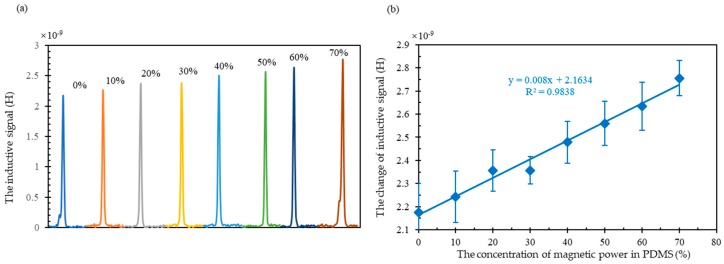
(**a**) the inductive signal (subtract the basic inductance value) for wear debris caused by coil with different concentration of magnetic powder range from 0 to 70%; (**b**) the relationship between the variation of inductive signal and the concentration of magnetic powder.

As shown in Figure 3, the signal of the same metal particle with 69.6 μm in diameter, would increase with increasing the concentration of the magnetic powder. The inductance signal variation is 2.174 nH when there is no magnetic powder in PDMS and the signal variation would increase to 2.755 nH when there is 70% magnetic powder in PDMS. The enhanced signal could be owed to the enhanced magnetic field. When the magnetic powder is introduced in the PDMS, the magnetic conductivity of PDMS is enhanced. So, the magnetomotive force, which is consumed by the material outside the coil, would decrease. With the same total magnetomotive force, the one act on the core of the coil would increase. As a result, the magnetic field inside the coil would be enhanced. Therefore, the induced inductive signal of the wear debris would also be enhanced.

As shown in Figure 3b, the variation of inductance signal is increasing linearly with the concentration of the magnetic powder increasing. The coefficient of determination was as high as 0.9838 which is acceptable. For a coil, the magnetic field of the core has a certain value. If we assume the magnetic powder concentration in supporter is 100% (which may be impossible in experiment), the magnetic field would be a value higher than the prior one. In our experiment, considering the concentration between them, the magnetic field would be a middle value. Consequently, the inductance signal, which is related to the magnetic field, followed the similar variation trend.

#### 3.2.2. The Detection Signal of Particles with Different Sizes

**Figure 4:**
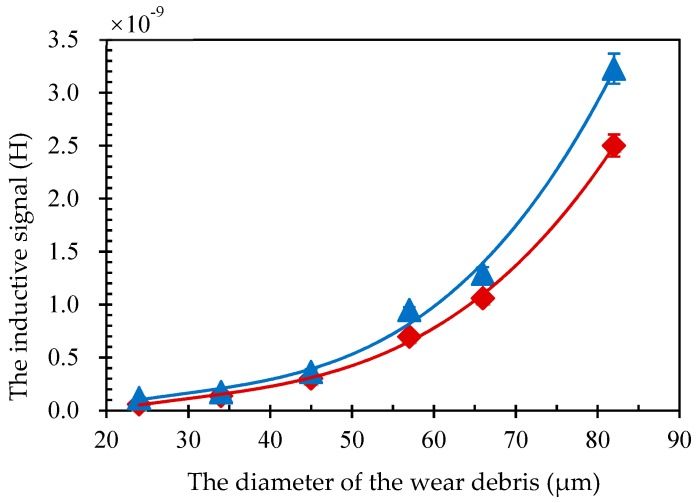
The variation of inductive signal of wear debris with different size for coil with 60% magnetic powder (blue triangle dot) and without (red diamond dot). The solid line is the fitting line of the experimental data.

The inductance signal of wear debris with different sizes was detected using a solenoid with and without magnetic powder. The diameters of the iron particles were 24 μm, 34 μm, 45 μm, 57 μm, 66 μm and 82 μm. As shown in Figure 4, the signal of wear debris is related to the cube of the particle size for both cases. This is consistent with previous research papers [27]. In addition, the signal of wear debris for coil with magnetic powder was larger than that without. These results further demonstrate the effectiveness of our method.

#### 3.2.3. Detection Limit Exploration of Magnetic Particle Enhancement

**Figure 5:**
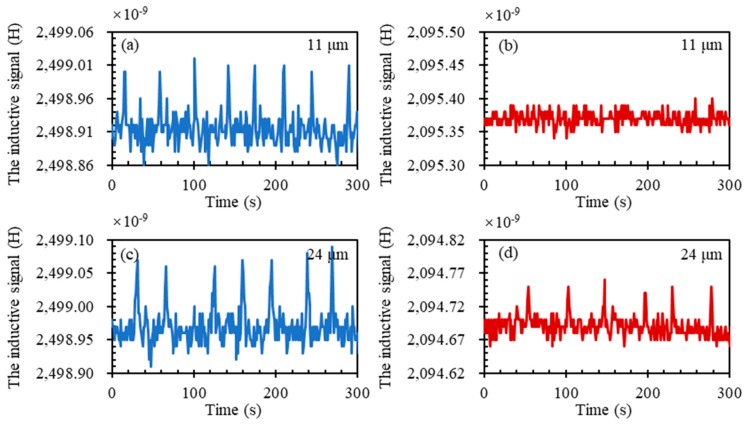
The inductive signal for 11 μm wear debris passing through coil with (**a**) and without (**b**) magnetic powder; the inductive signal for 24 μm wear debris passing through coil with (**c**) and without (**d**) magnetic powder.

To explore the detection limit of our sensor, metal particles with smaller diameters were used. As Figure 5 shows, the sensors with and without magnetic powder could detect 24 μm wear debris. However, the signal for sensors with magnetic powder was larger than that without. In addition, for 11 μm wear debris, the sensor with magnetic powder could detect while the sensor without could not. As shown in Table 2, we concluded the smallest size of the wear debris that can be detected in the reported papers of the past ten years. It is clear that the detection limit of our method has a great potential.

#### 3.2.4. The Oil Sample Experiment

**Figure 6:**
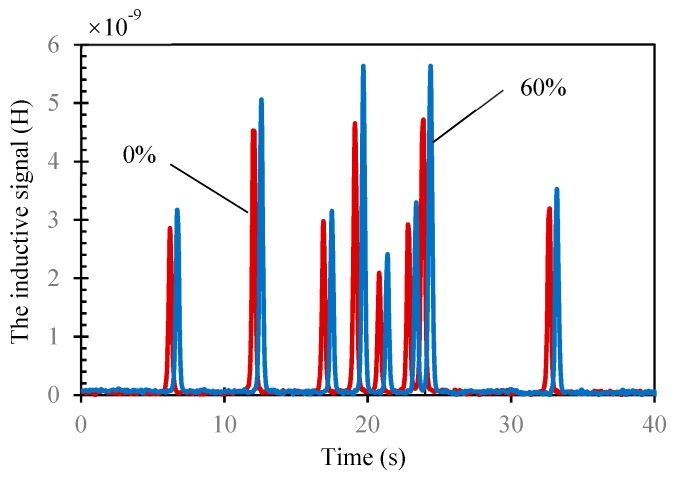
The inductive signal (subtract the basic inductance value) for 74–88 μm wear debris mixed with oil passing through coil with 60% magnetic powder (blue line) and without (red line).

As Figure 6 shows, when the lubricating oil mixed with wear debris passed through the two detecting coils (with 60% magnetic powder and without powder) in sequence, the signals were detected respectively. The signals with the blue line were the output of the coil with 60% magnetic powder while the ones with the red line were the output of the coil without magnetic powder. The two coils could detect the wear debris continuously. As the size of the wear debris was different, the amplitude of the detection signal was different. This is consistent with Section 3.2.2. In addition, the signals for every wear debris, detected by the coil with magnetic powder, were larger than that detected by coil without. Thus, our method could also work in practical work.

## 4. Conclusions

We proposed a novel method to improve the sensitivity of micro inductive sensors for wear debris detection. The method is very simple and maneuverable. Simply by surrounding some magnetic powder around the solenoid coil, it is found that the inductive signal of the wear debris would linearly improve with increasing the concentration of the magnetic powder. In addition, wear debris with 11 μm diameter could be detected using our method while it could not be done even by the same coil. However, the improvement range of our method is not high enough. We may combine this method with others to increase the detection sensitivity much more in future work.

## Figures and Tables

**Table 1:** The basic inductance of the chips used in the experiment.

Concentration	Inductance	Growth Rate
0%	2.118 μH	-
10%	2.182 μH	3.0%
20%	2.218 μH	4.7%
30%	2.301 μH	8.6%
40%	2.323 μH	9.7%
50%	2.384 μH	12.6%
60%	2.505 μH	18.3%
70%	2.740 μH	29.4%

**Table 2:** The smallest size of wear debris can be detected in references of the past ten years.

No.	The Smallest Wear Debris	Inner Diameter of Coil	Turns of Coil	Year	Ref.
1	11 μm	400 μm	50	2019	This article
2	33 μm	400 μm	- ^1^	2019	[14]
3	108 μm	300 μm	600	2019	[28]
4	134 μm	43 mm	-	2019	[27]
5	33 μm	900 μm	80	2018	[29]
6	40 μm	900 μm	20	2017	[15]
7	80 μm	900 μm	20	2017	[30]
8	20 μm	1000 μm	20	2013	[25]
9	50 μm	1300 μm	20	2011	[21]
10	50 μm	-	13	2010	[22]

^1^ “-” indicates that this parameter is not mentioned in the original reference.
